# Who Are the Most Influential Emergency Physicians on Twitter?

**DOI:** 10.5811/westjem.2016.11.31299

**Published:** 2017-01-19

**Authors:** Jeff Riddell, Alisha Brown, Ivor Kovic, Joshua Jauregui

**Affiliations:** * University of Washington, Division of Emergency Medicine, Seattle, Washington; † Ivor Medical, Leeds, United Kingdom

## Abstract

**Introduction:**

Twitter has recently gained popularity in emergency medicine (EM). Opinion leaders on Twitter have significant influence on the conversation and content, yet little is known about these opinion leaders. We aimed to describe a methodology to identify the most influential emergency physicians (EP) on Twitter and present a current list.

**Methods:**

We analyzed 2,234 English-language EPs on Twitter from a previously published list of Twitter accounts generated by a snowball sampling technique. Using NodeXL software, we performed a network analysis of these EPs and ranked them on three measures of influence: in-degree centrality, eigenvector centrality, and betweenness centrality. We analyzed the top 100 users in each of these three measures of influence and compiled a list of users found in the top 100 in all three measures.

**Results:**

Of the 300 total users identified by one of the measures of influence, there were 142 unique users. Of the 142 unique users, 61 users were in the top 100 on all three measures of influence. We identify these 61 users as the most influential EM Twitter users.

**Conclusion:**

We both describe a method for identifying the most influential users and provide a list of the 61 most influential EPs on Twitter as of January 1, 2016. This application of network science to the EM Twitter community can guide future research to better understand the networked global community of EM.

## INTRODUCTION

Twitter is an online social media platform that allows individuals to communicate through tweets. A tweet is an electronic message of 140 characters or less that is accessible to the public. By following other users, you can view their tweets in your personal timeline. Twitter is used by 23% of online adults, making it one of the most popular social media platforms globally. 1 In 2009 there were 672 emergency physicians (EP) on Twitter, and in January 2016 there were 2,234. 2,3 According to one survey, more than a quarter of emergency medicine (EM) faculty use Twitter. 4 Despite its popularity, some have called Twitter “untested” and argued that one must “learn who to follow and who to trust.” 5 Others have raised questions of relevance, threats to professionalism, and warned of rapid propagation of superficial and inaccurate information. 4, 6–8

### Importance

Dissemination of information on Twitter can be rapid and viral, and is heavily influenced by important opinion leaders. 9 Ideas flow from mass media to opinion leaders and then to the rest of a community. 10 Opinion leaders have a wide and loyal audience, have the power to influence the decisions of others, and disproportionately impact the spread and credibility of information. 11, 12 Opinion leaders on Twitter are the most followed and most connected. As such, they have the potential to influence the conversation and the content significantly more than their less influential counterparts. 9–11,12

Despite its popularity and potential pitfalls, there is a paucity of data examining influence among Twitter users in EM. Furthermore, existing measures of influence in social media are not directly applicable to Twitter. 13 The only existing measure of social media impact in EM is the Social Media Index (SMi). The SMi measures impact and quality of EM and critical care blogs and podcasts by measuring Google PageRanks, Alexa Ranks, Facebook Likes, Twitter Followers, and Google+ Followers. This measure was derived for a different purpose than ours. While useful for blogs and podcasts, it is a limited measure of influence specific to the Twitter platform, as it only includes total number of Twitter followers.

The influential group of opinion leaders in the EM Twitter community has not been defined. Defining this group is an important step toward understanding the spread of information among EPs on a social media platform.

### Goals of this investigation

We aimed to both describe a method to identify the most influential EPs on Twitter and present a current list. To perform this task we used *network science,* a new type of applied graph theory that incorporates several disciplines. 14 This list of Twitter influencers will help us better understand the intricate relationships of EPs on Twitter and lay the groundwork for future scientific inquiry. Demonstrating how this contemporary methodology of defining influence can be applied to Twitter will enable future application to other networks of EPs and advance understanding of those with local, national, and global influence.

## METHODS

This study was granted institutional review board exemption by the University of Washington Human Subjects Division.

### Data Gathering

Twitter lists are a common tool to group users into categories by various criteria. The first curated list of English-language EPs on Twitter was published in 2009. 2 Lulic and Kovic first developed their list by examining Twitter users’ biographies with web-based search tools from Twitter (www.Twitter.com), FollowerWonk (https://moz.com/followerwonk) and Twiangulate (http://twiangulate.com/search/). A snowball sampling technique was used to expand the list by exploring followers’ biographies and the Twitter accounts of organizations and journals related to EM. 15 The list is titled “Emergency Physicians” and is published by the Twitter user ↱@research_er. To the best of our knowledge, this is the most comprehensive list of EPs on Twitter.

From its January 2016 update, we gathered data about each member using NodeXL computer software (Microsoft Research, Redmond, WA). Variables including number of followers and tweets were recorded for each user.

### Data Analysis

Network science helps identify influential people based on several different metrics of influence. This is conceptually important because an individual may have social influence within a community for many different reasons. For example, an EP on Twitter may be influential because he or she has a large number of followers, has followers who are influential themselves, or has a unique group of followers to help disperse information. As such, sociologists have developed contemporary methods to identify influential members in a network and rank them according to different definitions of importance. These measures of importance are called centralities. 16 We used NodeXL and Gephi software (Gephi Consortium, USA) to perform network analysis and visualization. We measured influence of each user in the network by calculating in-degree centrality, eigenvector centrality, and betweenness centrality. 16

### Measures of Influence

#### In-Degree Centrality

Degree is a measure of connections based on the number of followers a user has *within a network*. In the case of our study, it is *not* the total number of followers a certain user has on Twitter. Instead, it is a measure of how many EPs are following a given user. In this measurement, each follower has equal weight.

Users with high in-degree centrality are considered to have prominence, prestige, and importance. 17 Users with a higher number of EPs following them have a higher capacity to effect the discussion among those users. It represents voices in the EM Twitter conversation that are likely to be listened to.

#### Eigenvector Centrality

Messages can spread broadly if retweeted, or passed along, by a few influential users. As such, being followed by one popular Twitter user bestows more influence than being followed by many brand-new Twitter users with few followers. Eigenvector centrality accounts for this by going beyond the number of followers a user has. It measures the collective influence of each follower. Being recognized by someone seen as powerful contributes heavily to one’s perceived influence. Eigenvector centrality elevates those users followed by a smaller, but more influential, number of followers. 18

#### Betweenness Centrality

Betweenness is a measure of information gatekeeping. Users with a high betweenness centrality provide the shortest paths between other users within the network. Because of their position within the network, they have considerable control over information diffusion. They are important in passing along information through a network. Users with high betweenness are frequently viewed as leaders. 19

### Outcomes

There is no single measure of importance that is paramount in understanding a social network. Rather, these centralities must be taken together to provide a robust measure of a user’s influence. 16 As such, we defined influence as being at the top of the list in all three measures of network centrality. We ranked the previously identified 2,234 EPs on Twitter by each of the three measures of influence. Users that appeared in the top 100 of all three measures of influence qualified as the most influential EPs on Twitter. We queried these users’ profiles for their name, gender, location, and year they joined Twitter.

## RESULTS

Of the 300 users in the top 100 of each measure of centrality (see Appendix), there were 142 unique users. Of the 142 unique users, 62 users appeared on all three lists. One of the 62 users was removed because it was the corporate account for a publication that could not be linked to a human physician. We identify the remaining 61 users as EM Twitter influencers (TIs).

Fifty-three of the 61 (87%) provide their full name in their profile. Of those whose gender was easily discernable from their profile, 9 of 59 (15%) are women. Seventy-one percent of TIs are located in the United States, with others in Europe (13%), Australia (9%), Canada (5%), and Costa Rica (2%). The earliest users joined Twitter in 2007, while the most recent influencer joined in 2014.

## DISCUSSION

The strengths of this study lie in a robust network analysis of over 2,200 EPs using three different measures of influence grounded in network science. We provide a network analysis method for determining the most influential EPs on Twitter. We also present a current list of those TIs, or Twitter influencers. This list helps quantify the qualitative concept of social influence and demonstrates a contemporary methodology for defining influence.

It is important to note that this analysis represents influence only *among emergency physicians,* and not broader influence among other healthcare networks or the lay public. For example, there are EPs with influence outside the EM community, like television star Travis Stork, MD, (@TravisStorkMD) who has 159,000 Twitter followers. He does not, however, influence the conversation or content among EPs because he is not followed by them and does not lie between them in the EP Twitter network.

Women make up a small percentage of the TIs. This gross disproportionality is consistent with other studies examining influential EPs. A recent study found that only 11% of academic chairs in EM are women. 20 Despite recent progress in gender equality, there remains considerable work to be done to improve equality for women, including in the realm of social media.

This work builds on Lulic and Kovic’s 2013 derivation of the EM users on Twitter list. 15 Without identifying users’ names, Lulic and Kovic presented the graphical data highlighting a small inner network of connected and influential EPs on Twitter. In this study, we provide a list of that influential inner network.

Our derived cohort had some overlap with the only other existing measure of social influence, the SMi. Of the 61 Twitter users affiliated with the top SMi blogs and podcasts, 41 (67%) were in our list of TIs. By applying several different, robust measures of influence, this curated list adds to our knowledge of the influential EPs on Twitter.

We believe this list of 61 TIs can be used as a valid foundation for future research around Twitter in EM. Rigorous analyses of the 61 TIs will move forward our understanding of the way Twitter is used for content, conversation, and professional development. For example, in-depth content analysis of the tweets of the 61 TIs would give insight into the EM subjects with the most weight on Twitter. A recent analysis of free open-access educational resources found imbalanced and incomplete coverage of EM core content. 21 Understanding the balance of content on Twitter may help EM practitioners and educators make informed decisions. Finally, and most importantly from a research perspective, analyzing the veracity of the content disseminated by the TIs would help further shine the light of evidence-based medicine on EM social media. The concerns about superficial and inaccurate information spreading would best be answered by analyzing the group most likely to influence the spread of information. This list should be used as a scholarly launching point to dive deeper into the conversation, content, and quality of the EM Twitter network.

In response to the concern that social media was gaining too much influence and that we are losing sight of key metrics of scientific value, such as citation indices, the satirical Kardashian Index was described in 2014. 22 This index is a direct proportion of number of Twitter followers to number of citations. With tongue firmly in cheek it urges caution with placing value on metrics of social media influence at the expense of more traditional metrics. It is important to bear in mind that the purpose of our study was to create a list that would help inform the community about the nature of social media influence as a whole rather than to create or elevate a celebrity culture around a few EPs. Nor does it confer any EM expertise. On the contrary, it is intended to focus our analytical lens on the TIs to give the greater EM community an understanding of how opinion is influenced and ideas are spread in this popular social network. This list is not intended to be an endorsement of these users or a metric of the quality of their messages. It is simply a measure of influence.

## LIMITATIONS

This study is limited to English-language speaking EPs. We did not contact the users to verify that they were EPs, though most of the 61 TIs are known to the authors as EPs. While our network analysis examined the number of followers for each user within the network of EPs, it did not analyze recent account activity for these users. It is possible that there are other influential EP users with high eigenvector, in-degree centrality and betweenness centrality who were excluded from our analysis because they have not been identified as EPs on the existing EPs Twitter list. This list is also limited to physicians and does not include those emergency medical services personnel, social workers, nurses, and pharmacists who are influential in the EM Twitter community.

## CONCLUSION

In summary, there is a growing network of EPs on Twitter, impacted by a small group of opinion leaders. To understand this network, we both describe a method for identifying the most influential users and provide a list of the 61 most influential EPs on Twitter as of January 1, 2016. This application of network science to the EM Twitter community can guide future research to better understand the networked global community of EM.

## Supplementary Information



## Figures and Tables

**Figure 1:**
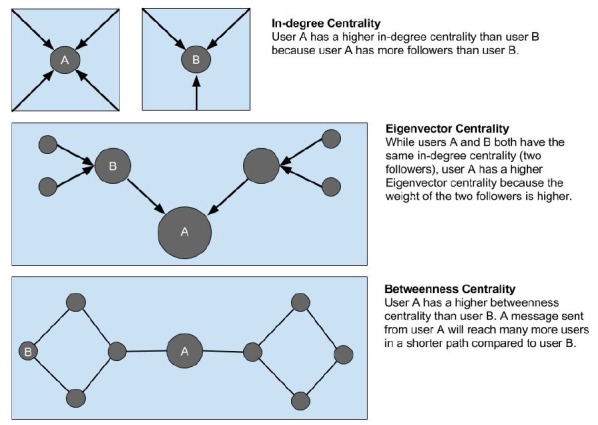
Pictorial description of In-degree, Eigenvector centrality and betweenness centrality.

**Figure 2:**
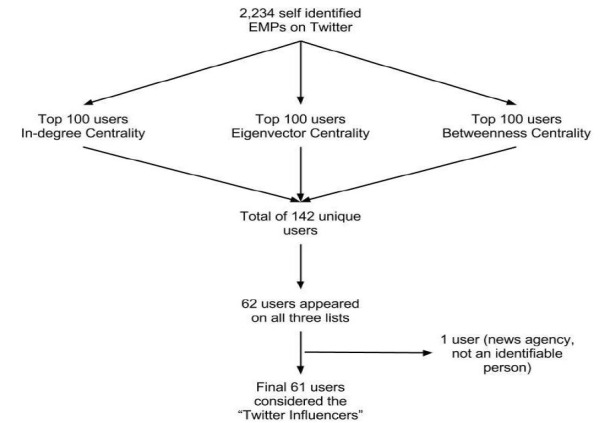
Study flow diagram. *EMP,* emergency medicine physicians.

**Table:** The most influential EM physicians on Twitter (as of 1-1-2016).

User	Twitter name	Gender	Location	Date joined
@_nmay	Natalie May	F	New South Wales, Australia	2012
@4hremergencydoc	4hremergencydoc	-	London, UK	2010
@airwaycam	Richard Levitan	M	New Hampshire, USA	2013
@amalmattu	Amal Mattu	M	Maryland, USA	2012
@andyneill	Andy Neill	M	Ireland	2011
@bobstuntz	EM Res Podcast	M	Pennsylvania, USA	2012
@brent_thoma	Brent Thoma	M	Saskatchewan, Canada	2012
@broomedocs	Casey Parker	M	Broome, NW Australia	2011
@cabreraerdr	Daniel Cabrera	M	Minnesota, USA	2014
@cliffreid	Cliff Reid	M	Sydney, Australia	2009
@criticalcarenow	Haney Mallemat	M	Baltimore, USA	2010
@drhowiemell	Dr. Howie Mell	M	North Carolina, USA	2012
@drjessepines	Jesse M. Pines, M.D.	M	Washington, DC	2011
@eleytherius	Michelle Johnston	F	Perth, Australia	2010
@em_educator	rob rogers	M	Kentucky, USA	2009
@embasic	Steve Carroll, DO	M	Texas, USA	2011
@emchatter	EMchatter	M	Missouri, USA	2012
@emcrit	Scott Weingart	M	New York, USA	2009
@emeducation	Rob Cooeny, MD, Med	M	Pennsylvania, USA	2008
@emergencypdx	Rob Orman	M	Colorado, USA	2010
@emergidoc	Kevin Kaluer DO, EJD	M	Tennessee, USA	2009
@emimdoc	David Marcus	M	New York, USA	2009
@emlitofnote	Ryan Radecki	M	Oregon, USA	2011
@emmanchester	Simon Carley	M	Manchester, UK	2009
@emswami	Anand Swaminathan	M	New York, USA	2013
@emupdates	reuben strayer	M	New York, USA	2011
@er_doc	ER doc	F	-	2008
@ercowboy	Pik Mukherji	M	New York, USA	2012
@grahamwalker	Graham Walker	M	California, USA	2007
@gruntdoc	GruntDoc	M	Texas, USA	2007
@jeremyfaust	jeremy faust	M	New York, USA	2009
@joelex5	Joe Lex	M	Pennsylvania, USA	2012
@ketaminh	Minh Le Cong	M	Queensland, Australia	2011
@klinelab	jeffrey kline	M	Indiana, USA	2014
@lwestafer	Lauren Westafer	F	New England, USA	2012
@m_lin	Michelle Lin	F	California, USA	2009
@mdaware	Seth Trueger	M	Illinois, USA	2011
@meganranney	Megan Ranney MD MPH	F	Rhode Island, USA	2011
@melherbert	EM:RAP’s Mel Herbert	M	California, USA	2008
@movinmeat	Liam Yore, MD	M	Pacifc NW, USA	2008
@nickgenes	Borborygmi	M	New York, USA	2008
@painfreeed	Sergey Motov	M	New York, USA	2013
@pedemmorsels	Sean M. Fox	M	North Carolina, USA	2011
@pemedpodcast	Andrew Sloas	M	Tennessee, USA	2011
@pharmertoxguy	Bryan D. Hayes	M	Maryland, USA	2012
@poisonreview	Leon Gussow	M	Illinois, USA	2009
@precordialthump	Chris Nickson	M	Melbourne, Australia	2008
@rainedoc	Todd Raine	M	British Columbia, Canada	2011
@rcempresident	Cliff Mann	M	London, UK	2010
@richardbody	Rick Body	M	Manchester, UK	2010
@rogerrdharris	Roger Harris	M	Sydney, Australia	2012
@sandnsurf	Mike Cadogan	M	Perth, Australia	2008
@smithecgblog	Stephen W. Smith	M	Minnesota, USA	2011
@socraticem	Victoria Brazil	F	Gold Coast, Australia	2011
@sonospot	Laleh Gharahbaghian	F	California, USA	2012
@srrezaie	Salim R. Rezaie	M	Texas, USA	2013
@takeokun	Jason T Nomura MD	M	East Coast, USA	2009
@tchanmd	Teresa Chan	F	Ontario, Canada	2009
@themattmak	Matt	M	London, UK	2011
@ultrasoundpod	Matt and Mike	M	Kentucky and Utah, USA	2011
@umanamd	Manrique Umana McD	M	San Jose, Costa Rica	2011

*M*, male; *F*, female.
