# Supplementary material for: Who Are the Most Influential Emergency Physicians on Twitter?
Source: West J Emerg Med. 2017 Jan 19;18(2):281–7. doi: 10.5811/westjem.2016.11.31299 (PMC5305138; doi:10.5811/westjem.2016.11.31299)
Supplement: Supplementary file 1 [file wjem-18-281-s001.pdf]

Appendix A: Most influential Twitter users ranked by different metrics of influence

In Degree

emcrit	1534
amalmattu	1389
melherbert	1292
m_lin	1228
emnews	1219
em_educator	1046
criticalcarenow	1014
emswami	1009
emergencypdx	994
precordialthump	967
sandnsurf	936
cliffreid	920
ultrasoundpod	915
joelex5	907
pharmertoxguy	873
srrezaie	842
mdaware	834
emmanchester	829
embasic	826
emlitofnote	803
ketaminh	768
airwaycam	746
emupdates	735
smithecgblog	716
poisonreview	650
emeducation	642
andyneill	635
klinelab	632
lwestafer	628
jeremyfaust	617
_nmay	599
eleytherius	573
brent_thoma	561
emimdoc	546
broomedocs	526
socraticem	513
grahamwalker	511
pemedpodcast	510

Betweeness

emnews	509924
emcrit	411571
melherbert	290817
amalmattu	288826
m_lin	180953
emmanchester	110094
rainedoc	108230
salahalem	107890
sandnsurf	105388
emswami	97109
em_educator	93804
mdaware	90376
criticalcarenow	87338
emergencypdx	86734
andyneill	79747
precordialthump	74465
cliffreid	73452
drangeloer	71180
joelex5	70877
ultrasoundpod	66899
srrezaie	62100
pharmertoxguy	61128
embasic	55567
ketaminh	45736
rcempresident	45708
airwaycam	41662
emlitofnote	40651
emupdates	39615
smithecgblog	38699
followbackdoc	37833
emeducation	35904
klinelab	30411
poisonreview	29659
umanamd	29196
gruntdoc	28555
4hremergencydoc	27973
jeremyfaust	24661

Eigenvector

emcrit	0.00401
amalmattu	0.00374
m_lin	0.00369
melherbert	0.00360
em_educator	0.00354
precordialthump	0.00341
criticalcarenow	0.00339
emswami	0.00339
joelex5	0.00330
emnews	0.00328
emergencypdx	0.00328
mdaware	0.00327
cliffreid	0.00326
emmanchester	0.00324
sandnsurf	0.00322
ultrasoundpod	0.00321
embasic	0.00312
pharmertoxguy	0.00309
emlitofnote	0.00306
srrezaie	0.00305
ketaminh	0.00299
emeducation	0.00299
andyneill	0.00291
emimdoc	0.00285
umanamd	0.00282
lwestafer	0.00281
emupdates	0.00281
jeremyfaust	0.00280
poisonreview	0.00276
airwaycam	0.00275
smithecgblog	0.00270
_nmay	0.00270
rainedoc	0.00265
brent_thoma	0.00264
eleytherius	0.00259
broomedocs	0.00252
klinelab	0.00248
bobstuntz	0.00247

umanamd	508
4hremergencydoc	503
bobstuntz	496
gruntdoc	494
rogerrdharris	484
drhowiemell	476
tchanmd	468
pedemmorsels	442
nickgenes	440
richardbody	430
rainedoc	423
rcempresident	399
emchatter	394
movinmeat	375
emdocbrett	374
painfreeed	374
johngreenwoodmd	372
takeokun	370
cabreraerdr	366
sonospot	364
edexam	362
emergidoc	362
drjessepines	356
ercowboy	349
humanfact0rz	341
em_resus	339
docbond007	335
jvrbntz	328
docib	326
emnerd_	321
emcardiac	311
fltdoc1	307
choo_ek	304
robjbryant13	299
meganranney	295
emtogether	294
thetechdoc	294
emo_daddy	287
themattmak	281
dreapadoirtas	280
er_doc	276
damian_roland	275

_nmay	24332
emimdoc	23912
eleytherius	23498
lwestafer	22696
adrianclark1	22302
beckybp	20303
brent_thoma	20050
aysabbagh	19919
drjessepines	18935
nickgenes	18679
emergidoc	18549
drhowiemell	17988
tchanmd	17615
grahamwalker	16341
sithlord2004	15712
bobstuntz	15444
kipschumacher	15341
pemedpodcast	15319
thetechdoc	14932
takeokun	14735
nightshiftmd	14374
ivorkovic	14256
socraticem	14092
cabreraerdr	13350
broomedocs	12619
insignaresemdoc	12303
pedemmorsels	12103
trainthetrainer	12084
ercowboy	11931
docnikko	11742
edwinleap	11690
rogerrdharris	11590
painfreeed	11504
movinmeat	11276
frontlinedoc	10836
richardbody	10758
istanbulemdoc	10509
pommalada	9804
xrawanx	9793
areddymd	9743
jord7an	9719
er_doc	9271

tchanmd	0.00236
4hremergencydoc	0.00231
ercowboy	0.00231
rogerrdharris	0.00231
grahamwalker	0.00229
socraticem	0.00228
painfreeed	0.00225
pemedpodcast	0.00223
gruntdoc	0.00222
movinmeat	0.00217
pedemmorsels	0.00216
cabreraerdr	0.00215
takeokun	0.00214
richardbody	0.00214
nickgenes	0.00204
drhowiemell	0.00204
jvrbntz	0.00200
rcempresident	0.00200
emdocbrett	0.00200
sonospot	0.00198
emchatter	0.00193
nickjohnsonmd	0.00193
docnikko	0.00192
johngreenwoodmd	0.00192
er_doc	0.00191
chrispartyka	0.00191
socalexmd	0.00191
edexam	0.00191
sluckettg	0.00189
robjbryant13	0.00188
ebmgonewild	0.00185
emtogether	0.00185
emnerd_	0.00184
global_em	0.00183
humanfact0rz	0.00182
emcardiac	0.00182
salahalem	0.00181
myemergencymed	0.00181
mfbellolio	0.00181
themattmak	0.00181
dreapadoirtas	0.00180
jcbeausoleilmd	0.00177

tessardavis	269	epnointoledo	9259	docib	0.00175
sherbino	265	sjdmd	9154	docbond007	0.00173
hawkmoonhems	262	meganranney	9019	drsamko	0.00173
sithlord2004	259	emchatter	8945	drfrankgabrin	0.00173
jojohaber	258	em_pearls	8716	jojohaber	0.00173
ebmgonewild	253	choo_ek	8687	drmehmettatli	0.00173
emjclub	253	patima	8584	emergidoc	0.00172
chrispartyka	247	adamcorley	8361	jploughrey	0.00171
annaemin5	243	themattmak	7728	kestlermd	0.00170
drrwinters	240	em_resus	7709	annaemin5	0.00168
drsamko	240	beren85	7663	shannonomac	0.00168
ermentor	239	teresawumd	7632	bodymender_n_ed	0.00166
realeddoc	236	nickjohnsonmd	7509	annecreaton	0.00166
drjfrank	231	global_em	7189	petrosoniak	0.00163
ultrasoundmd	229	spoedman	7187	beren85	0.00163
ergoddessmd	228	pepebravoc	7114	drjessepines	0.00162
poppaspearls	226	patarchambault	7060	pommalada	0.00162
kestlermd	222	bhanders	7003	meganranney	0.00162
areddymd	219	sonospot	6933	istanbulemdoc	0.00160
resusreview	217	emdocbrett	6518	bhanders	0.00160
		docbond007	6429		
